# Killing the competition: a theoretical framework for liver-stage malaria

**DOI:** 10.1098/rsob.210341

**Published:** 2022-03-30

**Authors:** Clemente F. Arias, Francisco J. Acosta, Cristina Fernandez-Arias

**Affiliations:** ^1^ Centro de Investigaciones Biológicas (CSIC), Madrid, Spain; ^2^ Grupo Interdisciplinar de Sistemas Complejos de Madrid, Spain; ^3^ Departamento de Ecología, Universidad Complutense de Madrid, Spain; ^4^ Departamento de Inmunología, Universidad Complutense de Madrid, Spain; ^5^ Instituto de Medicina Molecular, Universidade de Lisboa, Portugal

**Keywords:** *Plasmodium*, superinfection exclusion, liver-stage malaria, antimalarial vaccines, concomitant immunity

## Abstract

The first stage of malaria infections takes place inside the host's hepatocytes. Remarkably, *Plasmodium* parasites do not infect hepatocytes immediately after reaching the liver. Instead, they migrate through several hepatocytes before infecting their definitive host cells, thus increasing their chances of immune destruction. Considering that malaria can proceed normally without cell traversal, this is indeed a puzzling behaviour. In fact, the role of hepatocyte traversal remains unknown to date, implying that the current understanding of malaria is incomplete. In this work, we hypothesize that the parasites traverse hepatocytes to actively trigger an immune response in the host. This behaviour would be part of a strategy of superinfection exclusion aimed to reduce intraspecific competition during the blood stage of the infection. Based on this hypothesis, we formulate a comprehensive theory of liver-stage malaria that integrates all the available knowledge about the infection. The interest of this new paradigm is not merely theoretical. It highlights major issues in the current empirical approach to the study of *Plasmodium* and suggests new strategies to fight malaria.

## Graphical abstract

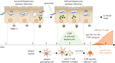

## 1. Introduction

The life cycle of *Plasmodium* parasites is an intricate journey through different tissues in their vertebrate and insect hosts. Malaria infections start with the inoculation of a few sporozoites under the skin by an infected female mosquito [1,2]. Sporozoites move through the dermis until they find a blood vessel and enter the bloodstream. Within 15 min, the first parasites leave the bite site and travel to the liver sinusoids [3]. After crossing the sinusoidal barrier, they infect hepatocytes [4,5] and differentiate into schizonts, syncytial cells that give rise to tens of thousands of haploid daughter cells known as merozoites [6,7]. Merozoites are eventually released into the blood [8], where they undergo several rounds of erythrocyte invasion and rupture until they mature into sexual gametocytes [9]. At this stage, they are ready to be taken up by a mosquito and leave their host.

When *Plasmodium* sporozoites reach the liver, they exhibit a perplexing behaviour: they do not infect hepatocytes immediately but migrate instead through the cytoplasm of several hepatocytes before settling down in their definitive host cells [10]. Although this behaviour was first observed *in vitro* [10], later studies confirmed that it also occurs *in vivo* [11]. Both mice- and human-infecting parasites [12] traverse hepatocytes, suggesting that this is a widespread feature among *Plasmodium* species. Hepatocyte traversal (HT) has puzzled researchers since its original description over 20 years ago [10]. To the best of our knowledge, the reasons for this behaviour remain unexplained to date. It was initially proposed that HT could be necessary for parasites to infect hepatocytes: the release of extracellular factors during HT might render nearby hepatocytes susceptible to infection [13,14]. Alternatively, HT might be needed to mature the molecular machinery used to enter the final host cell [11,15,16]. However, later studies with knockout sporozoites refuted this interpretation of HT [17,18]. Parasites with defects in *spect* or *spect2* genes cannot traverse host cells but they develop normally in the liver, showing that hepatocyte infection does not depend on HT [17–19].

A different approach to HT described it as a mechanism of immune evasion that could generate tolerance towards surface antigens of the parasite [20] or deceive activated CD8 T cells into targeting traversed hepatocytes instead of infected ones [21]. Again, Spect-defective sporozoites show that the parasite can normally evade the host’s immune response without traversing hepatocytes.

The existence of knockout sporozoites that do not traverse host cells and yet complete their life cycle implies that this behaviour is not essential for the parasite [22]. For this reason, although HT is acknowledged as the first step of the infection, it has come to be considered as an unimportant feature of liver-stage malaria and other aspects of the infection are usually interpreted as if they were independent of HT [23,24]. However, that the parasite does not need to traverse host cells does not mean that this behaviour is irrelevant for *Plasmodium*. Even if HT was actually superfluous, this should be rigorously proven before assuming that it does not affect other aspects of a parasite’s life. The lack of a satisfactory explanation for HT makes the current paradigm of liver-stage malaria necessarily incomplete.

In the absence of a comprehensive theoretical model of liver-stage malaria, sporozoites are widely considered as passive victims of the host’s immune defences, much like other intracellular pathogens such as viruses and bacteria. In this work, we show that this view is misleading because it fails to recognize the singularity of *Plasmodium* sporozoites. We hypothesize that their mode of action in the host's liver, radically different from that of viruses and bacteria, could allow them to manipulate the immune response of the host to their own benefit. Based on that hypothesis, we formulate an explanation of liver-stage malaria that accounts for all the known aspects of the infection. Crucial in this explanation is the behaviour of the parasite during HT. We show that a better understanding of the mechanisms used by the parasite to exploit the host suggests new lines of research in this field and points to possible drawbacks in the current approach to the development of antimalarial vaccines. It could also open the way for the finding of new strategies to fight the infection.

## 2. Subversion of host cells’ defences by *Plasmodium* sporozoites

*Plasmodium* sporozoites cross the sinusoidal barrier through the cytoplasm of Kupffer cells, professional phagocytes with a key defensive role. They remove a wide range of particles from the circulation, including bacteria that breach the intestinal barrier to invade the host’s tissues and other pathogens present in the blood [25]. In normal circumstances, micro-organisms captured by Kupffer cells are rapidly killed through a ‘respiratory burst’ that yields cytotoxic reactive oxygen species [26]. *Plasmodium* sporozoites block this mechanism and traverse the cytoplasm of Kupffer cells unharmed [2,5,9,27].

Once in the liver parenchyma, sporozoites infect hepatocytes using moving junctions (MJs), molecular structures similar to the tight junctions of mammal cells [28]. The MJ guides the invagination of the host’s plasma membrane to create a parasitophorous vacuole (PV) that will harbour the sporozoite during the liver stage of the infection [29]. The MJ acts as a molecular sieve that restricts the incorporation of key host proteins to the vacuolar membrane [30,31], thus precluding the fusion of the PV with lysosomes. This avoids the destruction of the parasite when infected cells detect and attack the nascent vacuole [32,33]. From the PV, the sporozoite controls the intracellular machinery of the hepatocyte through a membranous tubovesicular network that protrudes from the vacuolar membrane into the cell’s cytoplasm. This structure facilitates the import of nutrients and the export of waste products [33–35] and prevents the release of molecular clues that would attract immune cells towards infected hepatocytes [36,37].

The evidence presented above suggests that, as far as the host’s immune system is concerned, liver-stage malaria could well be a silent phase of the infection: traversed Kupffer cells do not respond to sporozoites crossing their cytoplasm, and although infected hepatocytes detect and attack the PV, they fall under the control of the parasite before they can react and alert the immune system. However, liver-stage malaria triggers a robust immune reaction that attracts both innate and adaptive to the site of the infection [38]. In the next section, we will show that this immune reaction is the host’s response to HT.

## 3. Hepatocyte traversal triggers an immune response in the host

The behaviour of *Plasmodium* sporozoites during HT is quite different from the one described above. To begin with, they do not conceal their presence in the cytoplasm of traversed cells. Parasites do not use MJs to traverse hepatocytes but transient vacuoles (TVs) that are rapidly digested by the host cell [31,39]. Sporozoites anticipate this reaction, using the drop in pH that accompanies digestion as a signal to leave the TV before its destruction [31]. Once in the cytoplasm of the traversed hepatocyte, the parasites move freely and breach the plasmatic membrane to egress the cell [31].

The wandering of the sporozoites across their cytoplasm and the rupture of their membrane are unambiguous signs of infection that could hardly pass unnoticed to traversed hepatocytes. Unsurprisingly, they react to these clues by launching a defensive response that involves (among other mechanisms) the NF-*κ*B pathway, a major inducer of inflammation [40]. It is certainly more remarkable that sporozoites usually repress this response in infected hepatocytes [37,40]. This means that the parasite can inhibit the NF-*κ*B pathway but it does not use this ability during this ability during HT, a puzzling behaviour for a pathogen.

The previous observations show that the activation of an immune response in traversed hepatocytes is not caused by the failure of the parasite to evade the host’s mechanisms of immune surveillance. On the contrary, it is the conspicuous migration of sporozoites across hepatocytes that facilitates their detection. Then, they tolerate in traversed hepatocytes the same inflammatory reaction inhibited a few minutes later in infected hepatocytes. The natural (albeit counterintuitive) conclusion of these observations is that migrating sporozoites compel traversed cells to initiate a systemic immune response in the liver.

Considering that the host’s immunity is typically lethal for pathogens, this behaviour would be apparently suicidal for the parasite. However, unlike other infections, the host’s immune system does not normally prevent the progression of liver-stage malaria [2,41,42]. Therefore, activating a systemic immune response during HT is not detrimental for migrating sporozoites. Quite the opposite: this response avoids concurrent malaria infections in the liver [43,44], which could be advantageous for the parasite. Preventing secondary infections in the host (superinfection exclusion or SE) is a widespread strategy among parasites and pathogens [45–48]. In intracellular parasites such as viruses and phages, SE is usually a cell-intrinsic mechanism (i.e. the protection against concurrent pathogens is restricted to already infected host cells [45]). In some plant viruses, the mechanisms of SE operate at the scale of the whole host organism and prevent the entry of new viruses in both infected and non-infected host cells [45]. This is also the case of helminths, in which an ongoing persistent infection can hinder the development of new larvae [49,50]. This phenomenon, sometimes called concomitant immunity, involves the release of cross-reactive antigens by adult worms that target larval structures, preventing the onset of secondary infections and limiting the size of the parasite population within the host [51].

We suggest that *Plasmodium* sporozoites could use a similar organism-level strategy to prevent secondary infections in the host. The parasite would use the host’s systemic response to kill secondary sporozoites in the liver. The conspicuous behaviour of the migrating sporozoites during HT would be intended to ensure the initiation of this response. In agreement with this assumption, co-transmission from a single mosquito is more frequent than superinfection by repeated mosquito bites in natural infections [52]. We propose that this strategy would be beneficial for the parasite because it would reduce within-host competition in the blood stage of the infection. A vast body of empirical evidence shows that competition among genetically unrelated strains affects the production of gametocytes [53,54] and decreases the probability of transmission for individual genotypes [55–61]. By neutralizing secondary infections in the liver, parasites with competitive disadvantage could prevent more competitive strains from reaching the host’s blood, thus increasing their chances of transmission. They would also reduce the likelihood of mixed infections, which could be more virulent than single infections [62] and compromise the survival of the host before the exit of the parasite.

Assuming a strategy of SE in *Plasmodium* would account for the role of HT in the context of the infection. It would also explain why this role is usually overlooked in the literature. Sporozoites that do not traverse hepatocytes might be unable to prevent concurrent malaria infections, but they would normally complete their life cycle in the host. The consequences of HT would only be evident in case of multiple infections, a situation that is not usually considered in experimental studies about HT.

From this approach, *Plasmodium* sporozoites would exhibit a dual relationship with the host’s immunity. The mechanisms of immune evasion described in the previous section would neutralize the host’s cell-level defences. This would avoid the destruction of the sporozoites by Kupffer cells and infected hepatocytes. For its part, HT would trigger a systemic immune response in the liver to prevent the onset of secondary malaria infections in the host. The interactions of the parasite with cell- and organism-level immunity are often confused in the literature. The parasite is widely assumed to inhibit the intracellular defences of Kupffer cells and infected hepatocytes to avoid a systemic response in the liver [63,64]. However, the absence of danger signals from Kupffer cells or infected hepatocytes cannot prevent traversed hepatocytes from triggering this response. Reciprocally, the detection of sporozoites by Kupffer cells or infected hepatocytes is not necessary to alert the host’s immune system. The behaviour of the parasite during HT would be sufficient to that end.

The potential use of the host’s immune response by the parasite sheds light on another puzzling aspect of malaria. A significant fraction of the sporozoites deposited by an infected mosquito do not reach the liver, remaining instead in the dermis or migrating to the lymph nodes that drain the site of inoculation [65,66]. Up to 50% of the parasites do not leave the dermis and some of them can even develop there and survive for weeks, although they do not contribute to erythrocyte infection in normal conditions [67,68]. Of the sporozoites that leave the site of the inoculation, around 30% migrate to nearby lymph nodes and are eventually degraded by dendritic cells [66,67].

Under the assumption of a strategy of SE in *Plasmodium*, these sporozoites could promote a local immune response in the bite site. The presence of activated immune cells would facilitate the immune detection of secondary sporozoites in those regions of the skin with a greater probability of infection (in humans, mosquito bites tend to concentrate in particular regions of the body [69]). In favour of this hypothesis, sporozoites display robust gliding motility and the ability to traverse cells in the skin [67,70]. Traversed cells in the dermis should alert the immune system, much as traversed hepatocytes do in the liver. Moreover, dendritic cells that detect the parasites in the lymph nodes activate CD8 T cells with affinity for sporozoite antigens [71].

## 4. Subversion of the host’s systemic immunity by *Plasmodium* sporozoites

Our hypothesis of HT as a mechanism of SE in *Plasmodium* relies on two key features of the host’s response to liver-stage malaria: its inability to stop primary infections and the protection it confers on the host against secondary malaria infections. The former implies that alerting the immune system during HT would not entail a high cost for the sporozoites. The latter suggests that it could actually benefit them. In this section, we will discuss the escape of primary parasites from the host’s systemic reaction. In the following section, we will analyse the mechanisms that neutralize secondary infections.

Liver-stage malaria triggers a robust immune reaction that leads to the activation of T cells [72]. Although sporozoites express hundreds of other genes, the dominant target of this response is usually the circumsporozoite protein (CSP) [37,73,74]. Among other functions, this protein participates in the recognition of host cells [75] and controls thousands of hepatocyte’s genes involved in metabolic processes crucial for parasite growth [37]. For this reason, the CSP is very abundant in traversed and infected hepatocytes in the early stages of the infection [76]. Since hepatocytes can present antigens secreted by the parasite into their cytosol [77], they end up displaying CSP antigens on their surface [21]. This, together with the immunodominance of these antigens, implies that most of the effector T cells recruited into the liver during the infection can identify both infected and traversed hepatocytes [21]. In spite of that, sporozoites normally evade T cell-mediated detection and progress into the blood stage [41,42].

The repeated ineffectiveness of the host’s T-cell response to kill *Plasmodium* sporozoites has been attributed to their short permanence in the liver. The parasite would leave the infected hepatocytes before T cells can detect and destroy them [4,42,78–81]. Apart from its short duration, liver-stage malaria would be similar to other intracellular infections. Given enough time, effector T cells would eventually identify and destroy all the infected hepatocytes, and hence the parasites [41,78].

In our opinion, this approach overlooks key aspects of the infection. Particularly, it does not take into account the singular mode of action of *Plasmodium* sporozoites. Unlike viral and bacterial infections, which involve multiple cycles of cell invasion and egress, liver-stage malaria requires just one round of cell infection. Once a parasite infects a hepatocyte, it remains inside the PV until it is ready to initiate the blood-stage of the infection [29]. Besides, migrating sporozoites infect their definitive host cells a few minutes after crossing the sinusoidal barrier [82], so most of the hepatocytes that will be parasitized are already infected within a few hours of the mosquito bite [66]. Owing to this mode of action, hepatocyte infection occurs long before the activation of naive T cells, which occurs around 24 h after the detection of the parasite [83,84]. The subsequent clonal expansion of activated T cells is only significant 24–48 h later [85,86]. Therefore, a notable delay exists between the entry of the parasites in their definitive host cells and the appearance of effector T cells in the liver. We suggest *Plasmodium* could use this delay to avoid T-cell-mediated detection.

**Figure 1.**
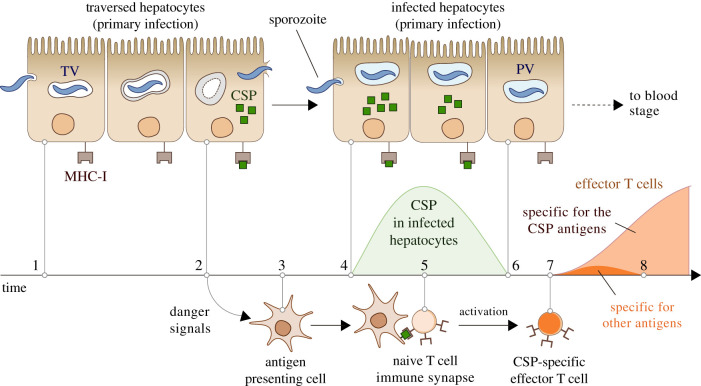
Hypothesized mechanism of *Plasmodium*’s immune evasion during liver-stage malaria. Hepatocyte traversal starts after the sporozoites cross the sinusoidal barrier (1). The digestion of the transient vacuole (TV) and the cell damage caused by migrating sporozoites trigger in traversed hepatocytes the release of danger signals and the initiation of an inflammatory reaction (2) that attracts innate immune cells to the site of the infection (3). To infect hepatocytes, the parasite makes extensive use of the CSP, which leads to a significant display of CSP antigens on the membrane of host cells (4). Simultaneously, innate immune cells start the activation of T-cell clones with affinity for those antigens, a process that involves the formation of immune synapses that may last several hours (5). When the parasite takes control of the infected cell, the levels of the CSP drop to undetectable levels (6). This occurs before the activation of T cells is complete (7). In the absence of CSP antigens on their membranes, infected host cells are effectively invisible to the CSP-specific effector T cells that reach the liver (8). The immunodominance of the CSP antigens would also diminish the expansion of T-cell clones with affinity for other non-dominant sporozoite antigens that might appear in later stages of the infection [93], further contributing to the invisibility of infected hepatocytes.

The dynamics of expression of the CSP antigens would be crucial in the immune evasion of the parasite. The levels of the CSP in infected hepatocytes are very high after the entry of the parasite [37,76,87–89], reaching peak expression at about 4–6 h post-infection in mice [77,90]. Afterwards, the parasite no longer synthesizes the CSP [91], which rapidly drops to undetectable levels [37,83,84,92]. Based on these observations, we suggest that infected hepatocytes would no longer display immunodominant antigens when effector T cells reach the liver. This would make them invisible to CSP-specific T cells (figure 1).

From this approach, the inefficiency of the adaptive response would not depend on the short duration of liver-stage malaria, as widely assumed in the literature [4,78–81]. Instead, it would result from the ability of the parasite to become undetectable to effector T cells by suppressing immunodominant antigens from the membrane infected hepatocytes. This strategy could also explain the immune evasion of the dormant phases (hypnozoites) present in some *Plasmodium* species. Hypnozoites persist in the liver over long periods of time and cause periodic reinfections of the host [94]. Clearly, their ability to escape T-cell-mediated detection cannot be explained by their rapid exit to the blood. Within the current paradigm, hypnozoites and sporozoites would rely on different and unexplained mechanisms of immune evasion.

## 5. *Plasmodium* sporozoites mimic a viral infection in the host’s liver to prevent secondary malaria infections

**Figure 2.**
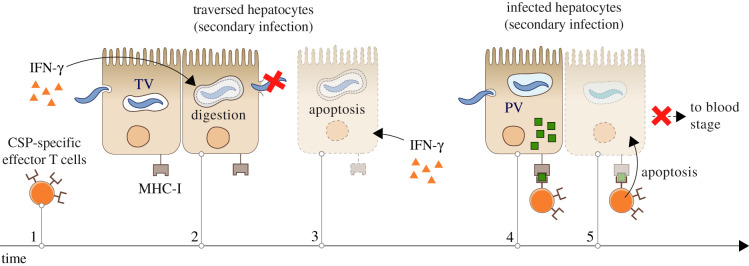
Hypothesized mechanism of immune susceptibility of secondary *Plasmodium* sporozoites. The immune reaction activated by a primary infection in the host’s liver results in the production of interferon (IFN)-*γ* and the activation of effector T cells with affinity for the CSP (1). IFN-*γ* activates the digestive machinery of traversed hepatocytes, increasing the risk of sporozoite destruction in the transient vacuole (TV) (2). The cell damage inflicted by migrating sporozoites could also lead to apoptosis in traversed hepatocytes, a reaction fostered by IFN-*γ* that would entail the death of the parasite (3). Some sporozoites may survive hepatocyte traversal (4) and infect hepatocytes. As occurs during primary infections, secondary parasites must use the CSP in the early stages of hepatocyte invasion, which leads to the large display of CSP antigens on the membrane of newly infected host cells (4). In contrast to primary infections, CSP-specific effector T cells are already present in the liver at this moment. This increases the probability of detection and destruction of infected hepatocytes before the disappearance of the CSP from their cytoplasm, which prevents the onset of the blood stage of the infection (5).

The same response that cannot stop primary malaria infections does normally protect the host from secondary infections [43,44]. This protection is largely mediated by interferon (IFN)-*γ* [44] and CSP-specific T cells [73,95,96]. The former, released by T cells and NKs, accelerates intracellular digestion in hepatocytes [78,91,97–101], increasing the risk for the parasite to be destroyed inside the TV [31,102]. IFN-*γ* could also stimulate apoptosis in traversed hepatocytes, which would entail the death of migrating sporozoites [103] As for effector T cells, it is remarkable that they can stop secondary infections but not primary ones. We suggest that this results from the impossibility of secondary parasites to implement the mechanism of immune evasion described in the previous section. Secondary parasites must also use the CSP in the early moments of the infection, which leads to the large display of immunodominant antigens in newly infected hepatocytes. In contrast to primary infections, activated CSP-specific T cells are already circulating in the liver at this stage of the secondary infection. This would greatly increase the chances of immune detection before the disappearance of the CSP from infected cells (figure 2).

The capacity of the host’s immune response to repeatedly neutralize secondary infections implies that IFN-*γ* and T cells must operate all across the liver. During liver-stage malaria, the ratio between infected and non-infected hepatocytes is in the order of 1 in 10^9^ in humans and 1 in 10^6^ in mice. A narrow immune response circumscribed to the sites of the infection would leave vast regions of the liver devoid of IFN-γ and CSP-specific effector T cells. Secondary sporozoites reaching these regions could safely traverse hepatocytes and suppress the display of the CSP in their host cells before being detected by effector T cells. In this scenario, secondary infections would be frequent. This is not the case in natural infections because the host’s response spreads throughout the liver. Key in the propagation of the response to primary infections is the type I IFN pathway [104,105]. In mice, IFN-α (a key element of this pathway) can be detected in the whole liver shortly after the bite by an infected female mosquito [106].

The type I IFN response creates a state of immune alert throughout the liver, minimizing the space where secondary infections can progress undetected. Assuming the existence of a strategy of SE in *Plasmodium*, this mechanism obviously suits the parasite’s interests, which raises an intriguing question: is the activation of the type I IFN pathway just a fortunate coincidence for *Plasmodium* sporozoites or is it somehow forced by the parasite?

This pathway is especially adapted to prevent intracellular infections that propagate through cell-to-cell transmission [107,108]. We have seen that this is not the mode of action of *Plasmodium* sporozoites, which infect a single hepatocyte and do not spread to adjacent host cells. Moreover, malaria infections start with the inoculation of a mean of 120 sporozoites in the skin [1,2] of which only 50% reach the liver [67], so very few hepatocytes are directly disrupted by the parasite. Therefore, the reasons for the host to trigger the type I IFN response during liver-stage malaria are far from trivial.

We hypothesize that sporozoites deceive the host into activating this pathway during HT. To do that, they would mimic the effects of a viral infection in the liver. Strong evidence in favour of this hypothesis is that they release double-stranded RNA (dsRNA) in the cytoplasm of hepatocytes [38,44,106,109]. Owing to the widespread use of dsRNA by viruses in genome replication [110], this molecule a clear indicator of viral infections [111–113]. Consequently, host cells react to its presence in their cytoplasm by activating the type I IFN response, which is a potent antiviral mechanism [114–117]. As should be expected, the detection of *Plasmodium* dsRNA by hepatocytes triggers this response during malaria [44,106,109]. Moreover, HT gives rise to a spatial pattern of immune alert that resembles the spreading of a virus in the liver. The cell damage caused by migrating parasites in traversed hepatocytes [118] and the release of dsRNA in their cytoplasm would create in the host the impression of an ongoing viral infection [119–121]. The activation and propagation of the type I IFN pathway would be the natural response to this stimulus [106,122,123].

Within our model of liver-stage malaria, the immunodominance of the CSP would also be crucial for the parasite’s strategy of SE (figure 2). By mimicking a viral infection in the liver, *Plasmodium* sporozoites could bias the adaptive response of the host towards the CSP antigens and ensure their immunodominance. Migrating parasites also release the CSP in the cytoplasm of traversed hepatocytes [21,41,124]. Its association with non-self dsRNA would induce traversed cells to process it as a viral protein, making it the target of the host’s adaptive immune response. Membrane vesicles decorated with the CSP have also been detected in the host’s liver during the infection [82]. It is tempting to speculate that the parasite could use these virus-like particles to provide additional clues of the propagation of a fake viral infection in the liver. This would further contribute to inducing a strong antiviral response against the CSP antigens.

## 6. Implications of the *Plasmodium*’s strategy of superinfection exclusion for the creation of anti-malarial vaccines

**Figure 3.**
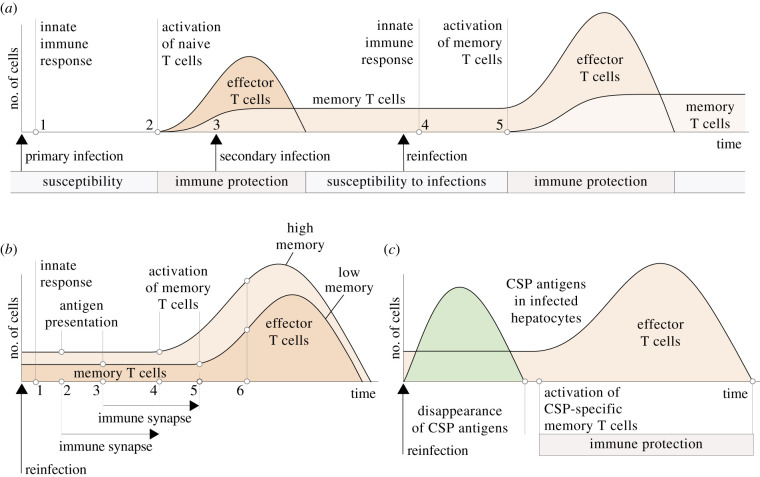
Dynamics of the interactions between *Plasmodium* sporozoites and the T-cell response of the host. (*a*) The presence of the parasite in the liver triggers an adaptive immune response (1) that leads to the activation of T cells that recognize sporozoite antigens (2). This response cannot stop the progression of the infection (figure 1) but prevents the establishment of secondary sporozoites in the liver (3) (figure 2). The immune protection provided by effector T cells disappears after clonal contraction, making the host vulnerable to new infections (reinfections). In case of reinfection (4), the adaptive response to sporozoites causes the activation of memory T cells in the liver (5). However, the host’s immune memory cannot neutralize the infection and avoid the onset of blood-stage malaria. (*b*) We hypothesize that the number of memory T cells does not improve the immune protection of the host. The immune reaction triggered by the sporozoites in the liver (1) attracts immune cells to the site of the infection. A greater number of memory T cells in the liver increases the rate of encounter with antigen presenting cells, accelerating the start of T-cell activation (2 versus 3). By contrast, the duration of the immune synapse (several hours) cannot be shortened by increasing the number of memory T cells (4 and 5). Thus, even if more memory T cells exhibit a greater clonal expansion (6), the appearance of effector T cells is necessarily delayed with respect to the entry of the parasite in the liver. (*c*) For this reason, infected hepatocytes could no longer display CSP antigens after memory T-cell activation. Therefore, activated memory T cells would not stop the reinfection even though they protect the host against future secondary infections.

Natural malaria infections do not endow the host with long-lasting immunity against *Plasmodium* sporozoites. Although repeated exposure to the parasites leads to the eventual formation of immunity against the blood-stage of the infection [125,126], long-term sterile immunity is never achieved [127]. Even if malaria infections normally result in the formation of memory T cells and antibodies that recognize specific sporozoite antigens, they cannot prevent successive reinfections of the host [42,128–131] (figure 3*a*). Similarly, the immune memory formed by antimalarial vaccines only provides a partial and temporary immunity that tends to disappear after a few months [42,132,133]. By way of example, the efficiency of the RTS,S/AS01 vaccine, the first vaccine against malaria to be tested in Phase 3, is around 36% over 4 years of follow-up [134,135]. Vaccines based on the use of irradiated sporozoites are more effective, reaching success rates close to 100% in the weeks or months that follow the inoculation of the parasites [127,136,137]. The efficacy of these vaccines is typically monitored for only a few months, so their long-term protection remains to be exhaustively evaluated [138]. However, studies in which vaccinated individuals are subject to successive challenges show that the results of the vaccines tend to worsen with time, which suggests that their protection would not be permanent [139,140].

The problem with natural infections and anti-malarial vaccines, as currently viewed, is that they induce the formation of too few long-term memory T cells. It has been suggested that the host is only protected if the number of memory T cells is above a critical threshold [133]. Accordingly, it is widely accepted that boosting the formation of memory T cells improves the performance of antimalarial vaccines [41,42,85].

A detailed analysis of T-cell activation suggests that this approach is overly simplistic. A greater number of memory T cells does not accelerate the encounter and processing of parasite’s proteins by antigen presenting cells (APCs) or the mechanism of T-cell activation, which involves the formation of immune synapses between T cells and APCs that persists for several hours [141]. Molecular signals delivered by the APC induce in the T cell the activation of hundreds of genes responsible for the acquisition of the effector phenotype and functionality [142]. It is unlikely that increasing the number of memory T cells could reduce the duration of this genetic program in each cell (figure 3*b*).

Therefore, there is a minimum delay in the activation of memory T cells that cannot be shortened by increasing the number of cells. We suggest that, as occurs with naive T cells, this delay could give the parasite an opportunity to suppress the display of key antigens by infected hepatocytes before the activation of memory T cells with affinity for those antigens. The immunodominance of the CSP during primary infections implies that most of the liver-resident memory T cells target this protein [129,130]. Now, the expression of the CSP drops to negligible levels in infected hepatocytes within a few hours of the entry of the parasite [37,83,84,92]. Even if the response of memory T cells is faster than that of naive T cells [143], memory T cells need at least 6 h to activate [85] and even longer to undergo significant clonal expansion [144]. During live-stage malaria, effector functions start to be detectable 24 h post-infection [84]. Based on the previous observations, we suggest that the timing of CSP expression relative to the activation of CSP-specific T cells could make the parasite invisible to the immune memory created by previous infections (figure 3*c*). This argument would also account for the ineffectiveness of T-cell-based vaccines against liver-stage malaria, which have traditionally targeted the CSP and other proteins used by the parasite in the early stages of the infection [145] (figure 3*c*).

**Figure 4.**
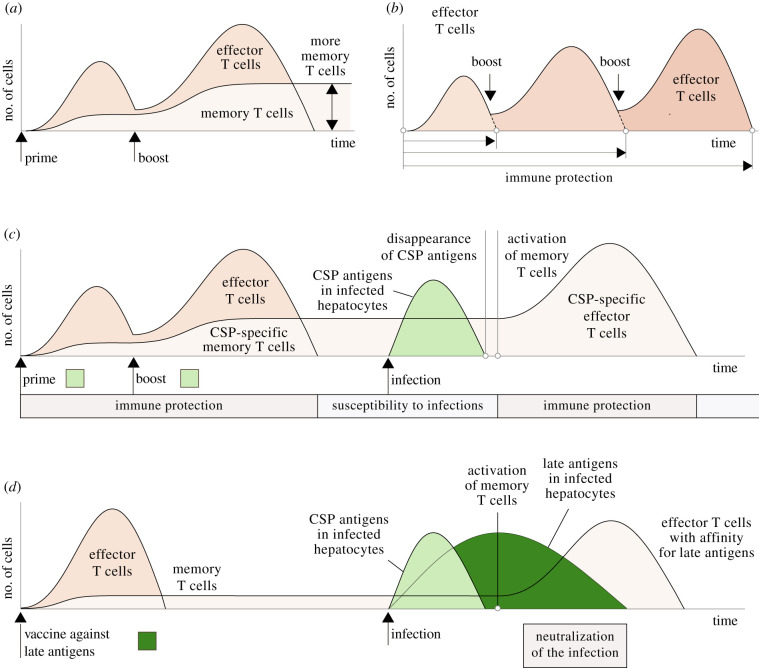
Hypothesized interactions between *Plasmodium* sporozoites and antimalarial vaccines. (*a*) In prime-boost protocols, a first dose of the vaccine triggers an immune reaction in the host and later inoculations boost this response, leading to the appearance of more memory T cells. (*b*) We hypothesize that the protection of prime-boost vaccines is not mediated by the formation of more memory T cells but by their effect on the duration of the T-cell response. Successive inoculations would extend the presence of effector T cells in the liver and consequently the protection against new infections (figure 2). (*c*) Vaccines targeting early antigens (those that disappear from infected hepatocytes within a few hours of the entry of the parasite) only confer the host with a transient immune protection. In the case of infection, those antigens are no longer present in infected hepatocytes after the activation of liver-resident memory T cells. This makes infected hepatocytes undetectable to the immune memory created by the vaccine. Activated memory T cells contribute to the strategy of superinfection exclusion of the parasite by preventing secondary infections in the host. (*d*) Targeting late antigens would protect the host against malaria infections. In this case, the activation of the memory T cells formed by the vaccine would coincide with the expression of target antigens on infected hepatocytes, increasing the probability of an effective neutralization of the infection.

The effect of T-cell-based vaccines can be improved by using prime-boost regimes in which a first inoculation (prime) triggers a T-cell response against the target antigens and successive inoculations (boosts) magnify this response [132]. Although this effect is normally attributed to the formation of more memory T cells [41,42] (figure 4*a*), we suggest that this does not explain the better results of prime-boost vaccines. The inoculation of periodic boosts also delays clonal contraction, extending the duration of the T-cell response and hence the protection against new infections (figure 4*a*). This would explain why frequent inoculations are needed to improve the degree of protection [146–148] (figure 4*b*). For instance, the RTS,S/AS01 vaccine is administered in three or four successive doses [149]. The vaccination with irradiated sporozoites usually requires multiple inoculations of thousands of defective parasites [150,151].

A corollary of the previous argument is that vaccines against early sporozoite antigens such as RTS, S vaccines (i.e. those that target antigens that disappear before the activation of memory T cells) would not operate as classical vaccines: their protection would depend on the presence of effector T cells in the liver and not on the activation of memory T cells in case of a new infection (figure 4*c*). This does not imply that these vaccines are ineffective but their protection would rely on a sustained T-cell response in the liver [78,147], which could entail potential costs for the host. A similar argument could be used to account for the action of vaccines consisting in the repeated inoculation of irradiated sporozoites. The short-term sterile protection provided by these vaccines could possibly be explained by the massive and sustained activation of T cells caused by the simultaneous arrival of thousands of parasites to the liver [83]. The long persistence of defective parasites in the liver could further contribute to extending the duration of this response [152].

The creation of an exceedingly large immune memory against *Plasmodium* sporozoites has other consequences that raise concerns about the use of prime-boost protocols against liver-stage malaria. The size of the pool of memory T cells is obviously finite, so the inclusion of new clones causes the loss of memory T cells with affinity for past infections. As a consequence, the diversity of the immune memory decreases, impairing the ability of the immune system to fight new pathogens in the future [153–155]. Saturating the pool of memory T cells with *Plasmodium*-specific clones is therefore a questionable strategy considering that malaria is not usually the only prevalent infection in endemic areas.

These issues could be avoided by targeting late sporozoite antigens since protection would be mediated in this case, as occurs with standard vaccines, by the activation of memory T cells (figure 4*d*). This approach is obviously constrained by the very existence of these antigens. Sporozoites might prevent the display of all of their antigens in infected hepatocytes after the first hours of the infection, making them virtually undetectable to memory T cells regardless of their target antigens. However, identifying potentially persistent antigens could prove a valuable line of research to create new and effective antimalarial vaccines [127,156]. Targeting the blood stage of the infection could provide effective strategies to fight malaria infections in endemic areas [157]. Recent studies in mice suggest that the use of antibodies with affinity for several epitopes of the CSP might also confer some degree of protection against liver-stage malaria, although their capacity to generate sterile protection *in vivo* remains to be evaluated [158].

## 7. Discussion

The empirical evidence gathered in the last decades has revealed stark contrasts between *Plasmodium* sporozoites and other intracellular pathogens. The singularity of liver-stage malaria is especially obvious in three features of the infection: first, sporozoites traverse several hepatocytes before invading their definitive host cells; second, each parasite infects a single hepatocyte and does not spread to adjacent host cells; finally, neither naive nor memory T cells can normally kill the parasite. Only secondary malaria infections (i.e. those that coincide with an ongoing primary infection) are susceptible to the host’s immune response [43,44].

These aspects of liver-stage malaria are often considered in the literature as unrelated to one another. In the absence of an accepted explanation for HT, it is impossible to ascertain its potential effects on later stages of the infection. In consequence, these effects have been excluded from the orthodox interpretations of liver-stage malaria to date. On the other hand, the infection of a single hepatocyte by each sporozoite is not considered determinant in the immune evasions of primary infections and reinfections, which are attributed instead to the short duration of the infection and the low number of liver-resident memory T cells, respectively. Under these assumptions, sporozoites would be passive victims of the host’s systemic response and only their rapid escape from the liver would save them from T-cell-mediated destruction [4,78–81].

In this work, we formulate an alternative model of liver-stage malaria in which the parasite manipulates the host’s systemic response to prevent concurrent infections. To do that, migrating sporozoites would simulate the spread of a virus in the liver to trigger an immune response in the host. The inhibition of secondary infections by the reaction to a primary infection is not extraordinary. The presence of effector T cells that can destroy infected host cells obviously increases the mortality of new pathogens that reach an already infected tissue. What makes liver-stage malaria different from other infections is that sporozoites remain inside the same host cell until the blood stage of their cycle. This establishes a clear-cut boundary between primary and secondary infections. Sporozoites could induce a systemic response and conceal themselves within their PVs while this response is still incipient. Then, they could take advantage of the delay in T-cell activation by suppressing the expression of key antigens in infected hepatocytes. By doing so, they would increase the vulnerability of secondary sporozoites without compromising their own survival. This strategy is not possible for typical intracellular pathogens that spread through cell-to-cell contagion and hence depend on the continuous infection of new host cells. In this case, primary pathogens are as exposed to the host’s immune response as secondary ones.

The existence of a strategy of SE in *Plasmodium* would pose new challenges to the experimental approach to liver-stage malaria. In natural infections only a few tens or hundreds of sporozoites reach the liver [1]. This situation stands in sharp contrast with experimental settings in which the infection is simulated by inoculating tens of thousands of sporozoites in the host. This is especially so for irradiated sporozoites, which have been used as a model of the infection in a number of malaria studies and as potential candidate antimalarial vaccines [159]. Irradiation does not affect the ability to traverse hepatocytes but prevents the onset of the blood-stage of the infection [160]. Rephrasing the Anna Karenina principle [161], all normal sporozoites are alike but each irradiated sporozoite is defective in its own way. Their behaviour in the liver can diverge in many different details from that of wild-type sporozoites. Now, the strategy of SE would depend on the precise execution of a strict sequence of steps during HT and infection. The failure to implement any of those steps by thousands of irradiated sporozoites would facilitate their massive destruction by Kupffer cells or hepatocytes. This could activate alternative immune pathways that are not normally triggered during natural infections [147,162–164], which would give confusing information about liver-stage malaria. For instance, the profile of cytokines secreted by Kupffer cells is different after the inoculation of irradiated and infectious parasites [165].

We do not deny the utility of these experiments. Quite the opposite, they provide valuable insights into many details of liver-stage malaria that would be impossible to obtain otherwise. Monitoring the progression of the parasite in a few hepatocytes is almost impossible, so using large numbers of wild-type or irradiated sporozoites could well be the only viable strategy to reveal key aspects of the infection. However, the differences between experimental and natural infections should be explicitly considered in the interpretation of empirical results.

Using the host’s immune response to prevent secondary infections in the host’s liver would be an optimal strategy for *Plasmodium*. First, even if the protection against secondary infections is not perfect, it would restrict within-host competition in the blood to strains that can been inoculated by infected mosquitos within a short time window [52]. This would facilitate the transmission of strains that would be otherwise outcompeted by more aggressive parasites. Second, the protection of the host would disappear with the clonal contraction of effector T cells, thus ensuring the availability of the host for future malaria reinfections. It cannot be ruled out that SE entails other benefits for the parasite. *Plasmodium* sporozoites have three sets of genes (nuclear, mitochondrial and plastid) and harbour occasional parasitic viruses [166,167]. These genomes could exhibit divergent interests, imposing further constraints on outbreeding and providing additional selective value for SE. Think, for instance, of the genetic incompatibilities created by *Wolbacchia* in its insect hosts [168] or by bacterial symbionts in some fungal species [169]. Regardless of its ultimate causes, the existence of a strategy of SE in *Plasmodium* would change our view of liver-stage malaria.

In this work, we formulate a coherent conceptual framework of the infection based on the hypothesis that the parasite uses the host’s immune system to kill potential competitors in the liver. This framework integrates all the available knowledge about liver-stage malaria, accounting in particular for the role of HT, a puzzling behaviour that remains unexplained with the current paradigm. The interest of an alternative view of liver-stage malaria is not merely theoretical. Our approach identifies unexpected critical limitations of the host’s immune system to neutralize *Plasmodium* sporozoites in the liver, which would explain the inefficiency of the antimalarial vaccines developed to date. Currently available vaccines would only replicate the parasite’s strategy of SE by targeting early sporozoite antigens. The protection conferred by these vaccines would depend on the presence of effector T cells in the liver. After their clonal contraction, the host’s immune memory would be unable to prevent the onset of future malaria infections. A better understanding of liver-stage malaria could prove useful to improve the efficacy of vaccines against liver-stage malaria. It could also suggest new strategies to interfere with the strategies used by the parasite to exploit the host.

## Data Availability

This article has no additional data.
